# Fold modulating function: bacterial toxins to functional amyloids

**DOI:** 10.3389/fmicb.2014.00401

**Published:** 2014-08-01

**Authors:** Adnan K. Syed, Blaise R. Boles

**Affiliations:** ^1^Department of Molecular Cellular and Developmental Biology, University of MichiganAnn Arbor, MI, USA; ^2^Department of Microbiology, Roy J. and Lucille A. Carver College of Medicine, University of IowaIowa City, IA, USA

**Keywords:** functional amyloid, bacterial toxin, biofilm, bifunctional protein, aggregation

## Abstract

Many bacteria produce cytolytic toxins that target host cells or other competing microbes. It is well known that environmental factors control toxin expression, however, recent work suggests that some bacteria manipulate the fold of these protein toxins to control their function. The β-sheet rich amyloid fold is a highly stable ordered aggregate that many toxins form in response to specific environmental conditions. When in the amyloid state, toxins become inert, losing the cytolytic activity they display in the soluble form. Emerging evidence suggest that some amyloids function as toxin storage systems until they are again needed, while other bacteria utilize amyloids as a structural matrix component of biofilms. This amyloid matrix component facilitates resistance to biofilm disruptive challenges. The bacterial amyloids discussed in this review reveal an elegant system where changes in protein fold and solubility dictate the function of proteins in response to the environment.

## AMYLOIDS

The secondary structure of the amyloid fold is one that is seen throughout life. Amyloids have long been studied because of their importance in human neurodegenerative diseases such as Alzheimer’s and Huntington’s diseases. There have been many reviews published on disease associated amyloids and they will not be discussed in this review (Chiti and Dobson, 2006; Eisenberg and Jucker, 2012). Amyloids were initially described in the 1850s as deposits in human tissues that stained with iodine, which was a characteristic of starch. A few years later though, it was shown that there were no carbohydrates in amyloid deposits but they consisted of proteins. Since then, amyloids have been found to be produced in many organisms and the biophysical and chemical properties of amyloids have been significantly investigated.

Amyloids are composed of fibrous oligomers of proteins that are characterized by a cross β-sheet structure running perpendicular to the fiber axis. The core of the amyloid fiber is made of protein backbones that form many hydrogen bonds between them leading to strong molecular forces. Because the strength of the amyloid is mainly independent of the side chains, proteins that can fold into amyloids do not have any sequence motifs, making it difficult to predict amyloid forming proteins. Even though they do not contain sequence homology, amyloids can be identified using biophysical properties including SDS-insolubility, protease resistance, and binding to the amyloid specific dyes Thioflavin T and Congo red (CR).

Amyloids have long been thought to be a result of protein misfolding, but over the past decade, this view has evolved to the understanding that some organisms utilize the amyloid fold for various functions aptly named Functional Amyloids (**Table 1**; Chapman et al., 2002). The most well studied functional amyloids are those made by bacteria that help form microbial communities called biofilms. These biofilms contain bacterial cells as well as matrix containing carbohydrates and proteins that hold them together affixed to a surface. Increasingly, it is being found that bacteria utilize the strength of the amyloid fold to make the strong biofilm matrix that resists disruption from stressors. As will be discuss below, many bacteria have developed toxin systems that are able to attack niche competitors or the host that can be abrogated by sequestering these toxins as amyloids where some have a second function in biofilm stability.

**Table 1:** Bacterial functional amyloids.

Organism	Protein	Functions	Reference
*Escherichia coli,* other *Enterobacteriaceae*	CsgA (Curli)	Biofilm matrix protein, surface attachment	Chapman et al. (2002)
*Pseudomonads*	FapC	Biofilm matrix protein	Dueholm et al. (2010, 2013)
*Mycobacterium tuberculosis*	MTP	Binding to human proteins and biofilm formation	Alteri et al. (2007), Ramsugit et al. (2013)
*Streptococcus mutans*	Adhesin P1 (antigen I/II, PAc)	Role in dental carries	Oli et al. (2012)
*Streptomyces coelicolor*	Chaplin	Hydrophobic coat for aerial hyphae	de Jong et al. (2009), Claessen et al. (2003)
*Staphylococcus aureus* and other* Staphylococci*	Phenol soluble modulins (PSMs)	Soluble virulence factor, biofilm dispersal/amyloid stabilizes biofilms	Wang et al. (2007), Schwartz et al. (2012)
*Klebsiella pnumoniae*	Microcin E492	Bacteriocin that is inactivated by amyloidogenesis	Biéler et al. (2005)
*Bacillus subtilis*	TasA	Soluble toxin/amyloid necessary for biofilm architecture	Stover and Driks (1999c), Romero et al. (2010)
*Listeria monocytogenes*	Listeriolysin O (LLO)	Forms pores in phagolysosome/inactive by pH shift in cytosol	Bavdek et al. (2012)
*Xanthamonas axonopodis*	HpaG (Harpins)	Triggers hypersensitive response in plants	Oh et al. (2007)

## AMYLOIDS AS STRUCTURAL MOLECULES

### CURLI

**FIGURE 1:**
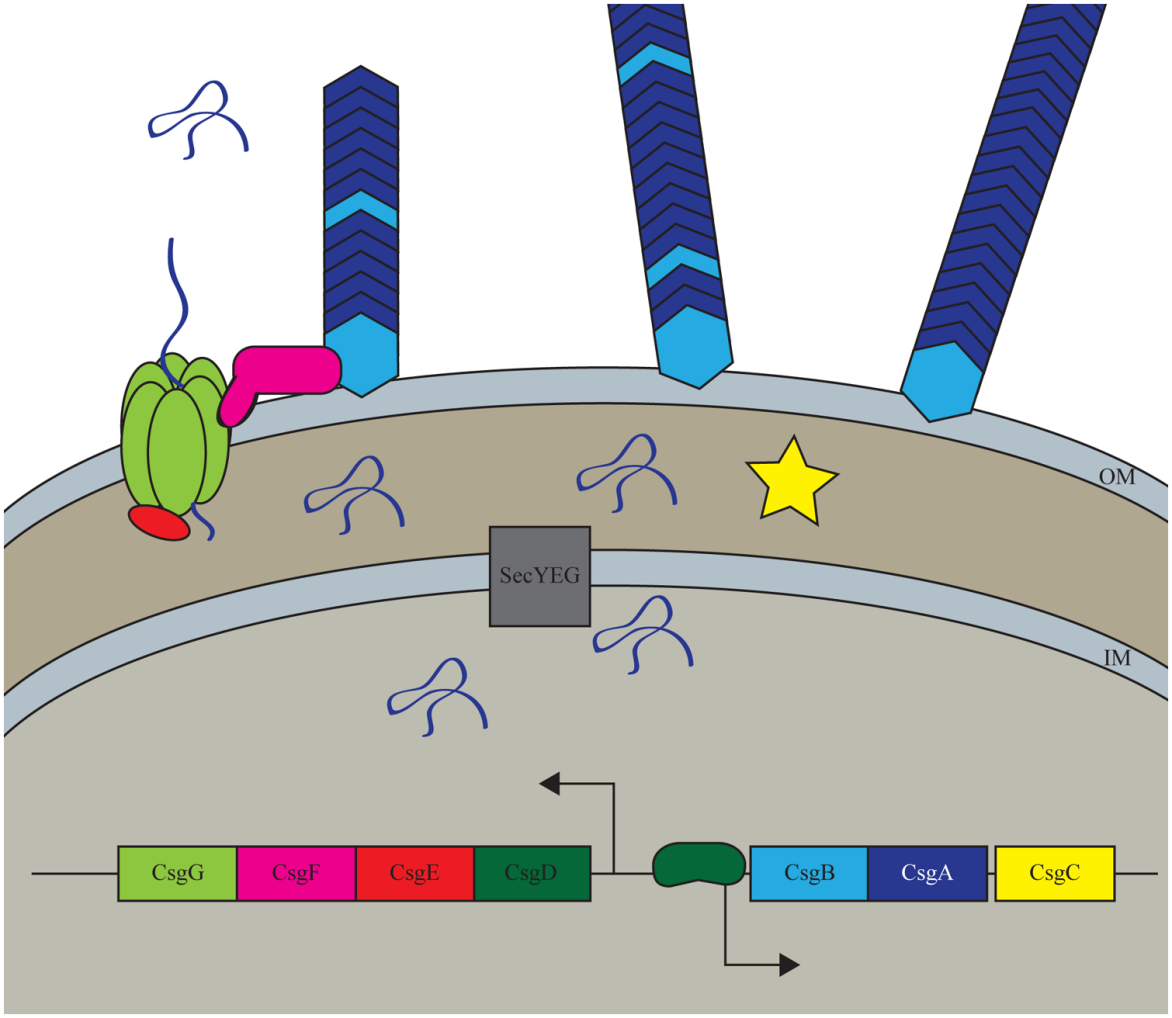
**Curli biogenesis model.** The curli system in *Escherichia coli* is a highly controlled process that only expressed the curli amyloid under conditions that promote biofilm formation. The system is transcriptionally controlled by the master regulator CsgD which increases the transcription of the major and minor subunits CsgA and CsgB. All Csg proteins other than CsgD are secreted through the Sec secretion pathway into the periplasm where CsgA, CsgB, and CsgF are then transclocated outside of the cell through the CsgG pore complex. CsgE and CsgF aid in proper export and localization of the structural components while CsgC has a less well understood role in the periplasm.

Curli are the most well studied bacterial functional amyloid. Through a dedicated pathway, curli form amyloids on the surface of *Enterobacteria*, such as *Escherichia coli,* and *Salmonella,* that aid bacteria in attaching to surfaces as well as defending the population from stress (Saldaña et al., 2009; Goulter-Thorsen et al., 2011; Zhou et al., 2012). Curli are made through a highly controlled master regulator CsgD, which induces the transcription of other curli specific genes (*csg*) to produce these amyloids (**Figure 1**; Brombacher et al., 2003). The major functional subunit of curli, CsgA, is secreted from the cell in a soluble form, leaving the outer-membrane through the pore formed by a hexamer of CsgG (**Figure 1**; Chapman et al., 2002; Robinson et al., 2006; Epstein et al., 2009). The minor fiber subunit CsgB is linked with the membrane and facilitates the nucleation of CsgA into amyloid fibers (**Figure 1**; Bian and Normark, 1997; Hammer et al., 2007). Proper assembly, localization, and regulation of curli fibers are modulated by CsgC, CsgE, and CsgF (Gibson et al., 2007; Nenninger et al., 2009, 2011; Evans and Chapman, 2014). Not only are the curli genes under strict genetic regulation, but it has been shown that cellular chaperones can modulate the fold of CsgA to prevent improper folding in the cells (Evans et al., 2011).

Curli fibers are important for *E. coli* surface colonization and biofilm formation (Chapman et al., 2002; Saldaña et al., 2009; Crémet et al., 2013; DePas et al., 2013; Giaouris et al., 2013). The expression of curli is a tightly regulated process in regards to the environment around the bacteria as well as within a biofilms community. Recently, it has been shown that there is spatial regulation within an *E. coli* rugose biofilms where curli producing cells are localized to the exterior of the biofilms, whereas cells on the interior of the community were not producing curli fibers (DePas et al., 2013; Serra et al., 2013). This bimodal growth allows for a protective shell of matrix-encased cells that contain a population of cells that ready to disperse and disseminate when conditions become favorable.

### OTHER FUNCTIONAL AMYLOIDS PRODUCED BY BACTERIA

Emerging evidence suggest that amyloids likely play a structural role in some naturally occurring environmental biofilms. Recent work utilizing conformational antibodies that specifically bind to the amyloid fold, and the amyloid-specific dye thioflavin-T, provide evidence of amyloids being present in biofilm samples for fresh water lakes, drinking water, and activated sludge from a water treatment facility (Larsen et al., 2007). The bacteria present in these biofilms include representatives from Actinobacteria, Bacteroidetes, Chloroflexi, and Proteobacteria. Further studies revealed one member of this community, *Pseudomonas fluorescens*, was able to produce an amyloid found in the biofilm matrix (Dueholm et al., 2010). Proteomic analysis revealed the major subunit of the amyloid to consist of a protein named FapC (Dueholm et al., 2010). The genes necessary for formation of this amyloid were traced to the *fapA-F* operon, which is conserved in many *Pseudomonas* species. FapC contains repeat motifs and conserved Asn/Gln consensus residues similar to curli and the prion and spider silk amyloid proteins (Dueholm et al., 2010). Further studies have demonstrated that other *Pseudomonads* also form Fap fibrils that result in biofilm formation (Dueholm et al., 2013). These finding suggest functional amyloids are likely abundant in naturally occurring biofilms consisting of diverse microbial members.

The pathogens *Mycobacterium tuberculosis* and *Streptococcus mutans* have also been found to produce functional amyloids. In the case of *M. tuberculosis*, thin, aggregative flexible pili, named MTP, were observed during human infection (Alteri et al., 2007). These pili possess biophysical and morphological characteristics of amyloids and bind to the human extracellular matrix component, laminin. Proteomic analysis suggests the structural subunit of MTP is a proteolytically processed version of a 10.5 kDa protein encoded by the open reading frame Rv3312A (*mtp*) in *M. tuberculosis* strain H37Rv (Alteri et al., 2007). In addition, serum from tuberculosis patients contained antibodies that specifically recognized MTP, suggesting a role for MTP during infection (Alteri et al., 2007). MTP was also found to be important in the formation of biofilms by *M. tuberculosis* (Ramsugit et al., 2013). *S. mutans* is a member of the oral microbiome and is linked to the disease dental caries because of it’s ability to produce acid from the utilization of dietary sugars. Recent work suggests that the *S. mutans* adhesin P1 (antigen I/II, PAc) is an amyloid-forming protein (Oli et al., 2012). During biofilm growth *S. mutans* displayed amyloid fibers as evidenced by transmission electron microscopy, bound the amyloidophilic dyes CR and Thioflavin T (ThT), and possessed green birefringent properties of CR-stained protein aggregates when viewed under cross-polarized light (Oli et al., 2012). Importantly, human dental plaques contain microbial amyloids, suggesting a role for this protein fold in dental carries (Oli et al., 2012).

Chaplins are a class of hydrophobic proteins that spontaneously self-assemble into amyloid fibrils (Claessen et al., 2003). The spore-forming filamentous bacterium *S. coelicolor* uses chaplin amyloids to complete its lifecycle progression (Claessen et al., 2003). Under starvation conditions *S. coelicolor* produces aerial hyphae that extend upward out of the soil. Spores are produced in these hyphae and they are release once the soil surface has been breached. Vegetative *S. coelicolor* cell surfaces are hydrophilic, so to break the soil/air interface, the cells must first develop a hydrophobic coat. To this end, *S. coelicolor* secretes monomeric chaplin proteins (encoded by *chpA-H*; Claessen et al., 2003; Elliot et al., 2003). These hydrophobic proteins have been shown to form β-sheet rich amyloid fibers on contact with air, therefore chaplin amyloids are essential for *S. coelicolor* to complete its lifecycle from vegetative cells to spore containing hyphae.

## BIFUNCTIONAL PROTEINS

### PHENOL SOLUBLE MODULINS

Phenol soluble modulins (PSMs) are a family of proteins that are found in Staphylococci, most notably the significant human pathogen *Staphylococcus aureus* and the human commensal Staphylococcus epidermidis (Mehlin et al., 1999; Wang et al., 2007). *S. aureus* has nine characterized PSM peptides that are all regulated by the accessory gene regulator (AGR) quorum sensing system (Janzon et al., 1989; Wang et al., 2007). There are four PSMα, two PSMβ, and δ-toxin that are present in three separate regions of the chromosome. The newest member to this family is the *N*-terminal signal sequence of the AgrD molecule *N*-AgrD (Schwartz et al., 2014). This sequence is critical for localization of the propeptide to the membrane and once cleaved from the rest of the AgrD molecule has many structural and functional similarities to the other PSMs (Schwartz et al., 2014). In addition, some stains of *S. aureus* contain a pathogenicity island that harbors an ninth PSM called PSM-mec (Queck et al., 2009). The PSMs are secreted from the cells by a dedicated, essential secretion system called phenol-soluble modulin transporter (PMT; Chatterjee et al., 2013). These PSM peptides are amphipathic α-helices, meaning that one face of the helix in hydrophobic while the other is hydrophilic (Wang et al., 2007). This shared property is thought to allow for them to form pores in the membranes of competing microbes and host cells to invade tissues and evade immune cells.

**FIGURE 2:**
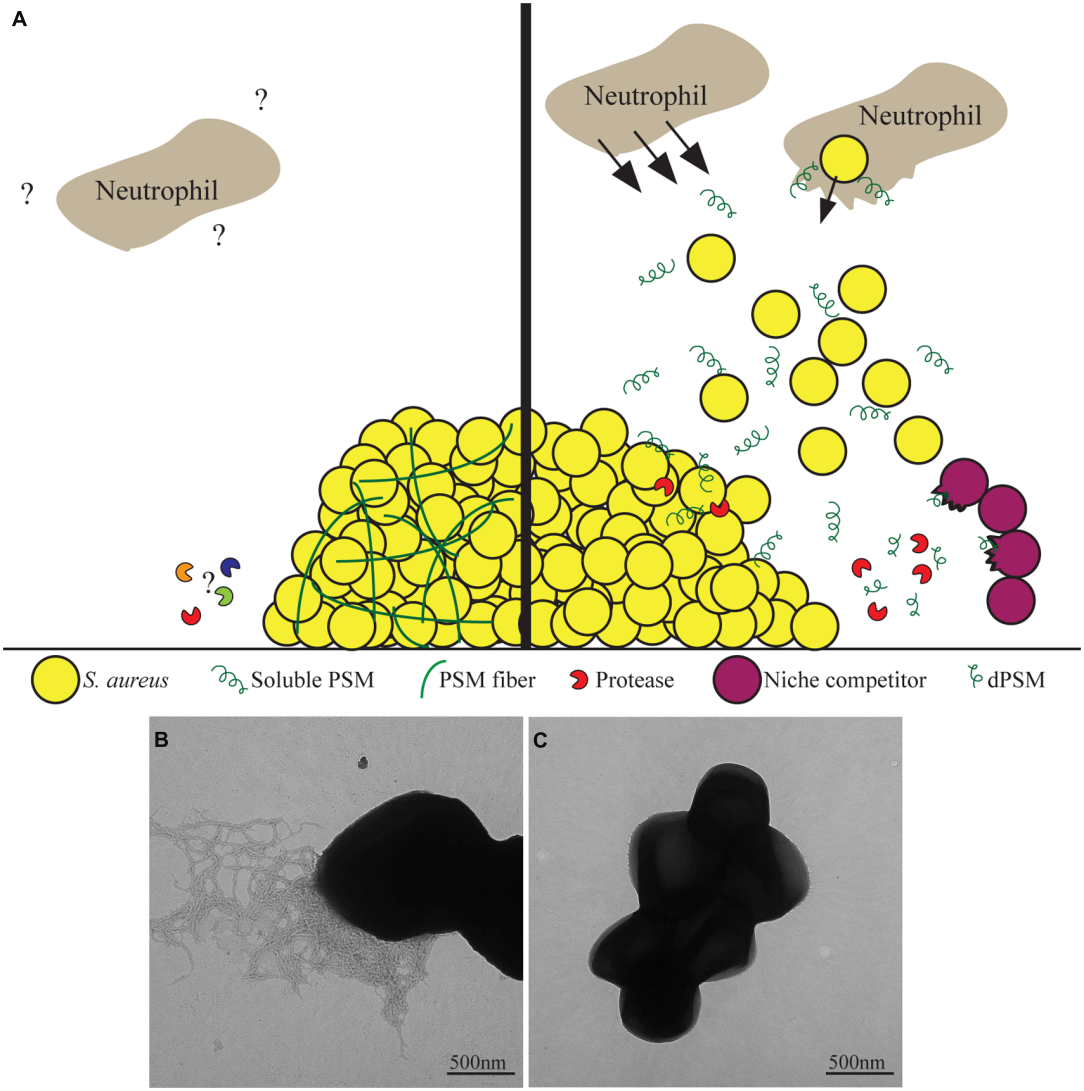
**Phenol soluble modulins as bifunctional proteins.** **(A)** *Staphylococcus aureus* produces Phenol soluble modulins (PSM) proteins that have been found to have many diverse functions. As soluble molecules, they are able to stimulate chemotaxis of neutrophils as well as aid *S. aureus* escape from the phagolysosome upon phagocytosis. Additionally, soluble PSMs can disperse biofilms as well as be proteolytically processed into PSM derivatives of phenol-soluble modulins (dPSMs) that can kill niche competing bacteria. Once polymerized into amyloid fibers, PSMs provide functional support to the biofilm community that prevents degradation by biofilm dispersing enzymes. The interactions between PSM fibers and immune cells have yet to be characterized. **(B)** Transmission electron microscopy of a *S. aureus* biofilm that is producing PSM amyloids fibers. **(C)** *S. aureus* biofilm cells under conditions where amyloid fibers are not detected.

Phenol soluble modulins have been shown to be critical determinants of *S. aureus* to cause skin abscesses and wounds in murine models as well as aiding in the survival of *S. aureus* in murine bacteremia models (Wang et al., 2007). PSMs stimulate neutrophil chemotaxis through the human formyl peptide receptor 2 (FPR2), at nanomolar concentrations, independent of the formylation state of the peptides (**Figure 2**; Kretschmer et al., 2010). Once the neutrophils are in close proximity, PSMs are able to infiltrate cells and cause cell death (**Figure 2**; Wang et al., 2007). Recently though, the field has shifted toward the hypothesis that in the host, PSMs may be important in virulence once *S. aureus* is phagocytosed by neutrophils (Surewaard et al., 2012). This hypothesis is supported by data demonstrating the serum lipoproteins are able to bind to and inactivate PSMs, meaning that they would be unable to function in the presence of serum in the host (Surewaard et al., 2012). Secondly, once phagocytosed by neutrophils, *S. aureus* cells highly upregulate the production of PSM peptides which aid in escaping from the phagolysosome (**Figure 2**; Surewaard et al., 2012).

Phenol soluble modulins are not only reported to be important for *S. aureus* pathogenesis and virulence against the host. PSMs have also been shown to be antimicrobial against potential competitors. PSMs were first determined to have antimicrobial effects from *S. epidermidis* (Cogen et al., 2010). PSMs share structural similarity to mammalian antimicrobial peptides such as LL-37, thus it was tested to determine if *S. epidermidis* PSMs were able to kill mammalian pathogens (Cogen et al., 2010). Two PSMs from *S. epidermidis* were found to have antimicrobial effects against *S. aureus* as well as group A *Streptococcus* (GAS) and worked in conjunction with LL-37 to have synergy (Cogen et al., 2010). Focus then turned to determine if and how *S. aureus* PSMs may act against niche competing bacteria. It was found that full length PSM peptides possessed little antimicrobial activity but derivatives of PSMs (dPSMs), PSMs that have been proteolytically processed to be missing the first few amino acids, have strong antimicrobial properties against *S. pyrogenes*, *S. epidermidis*, and GAS (**Figure 2**; Joo et al., 2011; Gonzalez et al., 2012). Furthermore, when a colony of *S. aureus* is grown in close proximity to *S. epidermidis* or GAS, dPSMs are localized to the zone of inhibition of the competing bacteria (Gonzalez et al., 2012).

Along with their role as toxins, the biophysical properties of PSMs give them several unique properties in modulating *S. aureus* communities. First these are the ability of *S. aureus* PSMα and PSMβ to facilitate dissemination and spreading of a colony over soft agar plates (Tsompanidou et al., 2011). This suggests that some PSMs are able to act with surfactant-like properties lowering hydropathy and allowing for *S. aureus* to spread (Tsompanidou et al., 2013). Additionally, PSMs have been shown to be important for the formation of biofilms. The PSMs are important biosurfactants that aid in the characteristic waves of dissemination of parts of the biofilms to colonize other areas (**Figure 2**; Periasamy et al., 2012).

Along with these properties attributed to the soluble PSM peptides, they are able to form amyloid fibers which stabilize *S. aureus* biofilms (Schwartz et al., 2012). This switch changes the soluble α-helical peptides into β-sheet rich protein aggregates (Schwartz et al., 2012). This aggregation, like other amyloids, is through a self-templating mechanism that facilitates the transformation of other nearby proteins to adopt this amyloid fold. The PSMs formed amyloid fibers in biofilms that were grown in a non-standard rich media containing peptone, glucose, and NaCl. These biofilms were completely resistant to known biofilm dispersing enzymes Proteinase K, DNase, and Dispersin B, suggesting that the PSM amyloids are able to structurally stabilize the biofilm against enzymatic targeting of the previously characterized matrix components (**Figure 2**; Schwartz et al., 2012). Importantly, PSMs were demonstrated to have bifunctional abilities to either strengthen biofilms or disperse them dependent on their secondary structure (**Figure 2**). If monomeric PSMs were added to an established biofilm they exhibits surfactant like properties, dispersing the biofilms in a concentration dependent manner, whereas PSM fiber addition does not disperse biofilms (Schwartz et al., 2012).

Further studies are needed to investigate the flexibility of the PSM peptides to switch from soluble peptides to amyloid fibers and if this change is irreversible or only temporary. Interestingly, where PSMα and PSMβ peptides were shown to be essential for *S. aureus* colony spreading, it has been shown that colony spreading can be inhibited by δ-toxin (Omae et al., 2012). This may suggest a role in amyloid nucleation by δ-toxin on the other PSMs that inhibit their ability to act as surfactants. It is tempting to speculate that these fibers may be reservoirs of toxins that *S. aureus* can utilize to both defend itself while also causing the population to disseminate and escape. It would also be interesting to see if the aggregation of these peptides into amyloids fibers is able to abrogate neutrophil chemotaxis thus acting as a way to hide from the immune system when forming biofilms in the host. Much more work is needed to fully understand how *S. aureus* and other Staphylococcal species utilize the fold of these PSM peptides to modulate their function.

### MICROCIN E492

Microcin E492 (MccE492) is part of a large family of bacteriocins that are antimicrobials secreted by bacteria to kill niche competitors. In general, bacteriocins are pore-forming proteins that kill competitor microbes by forming pores in their membranes, decreasing membrane potential (de Lorenzo and Pugsley, 1985). MccE492 is produced by Klebsiella pnumoniae that is able to target many *Enterobacteria* species such as *E. coli*, and *Salmonella* (de Lorenzo and Pugsley, 1985; Destoumieux-Garzón et al., 2003). MccE492 is found as both an unmodified peptide as well as a posttranslationally modified molecule with a catechol-type siderophore molecule (Thomas et al., 2004). This post translational modification allows for microcin to be recognized by siderophore catecholate receptors of target organisms that cause an uptake of the mature MccE492 molecule into the periplasm (Destoumieux-Garzón et al., 2003). The exact mechanism of cell death is unknown due to the fact that there needs to be much more MccE492 present for antimicrobial activity compared to the amount needed for membrane permeabilization (Destoumieux-Garzón et al., 2003).

Microcin E492 is unique to known microcins in that it is produced through exponential and stationary phase, whereas other microcins are only produced in stationary phase. Interestingly, MccE492 loses its antimicrobial activity when the population enters stationary phase even though the protein is still present at high levels (Corsini et al., 2002). This observation led to the discovery that in stationary phase, MccE492 aggregates to form amyloid fibers (Biéler et al., 2005). Aggregation into an amyloid abolishes the toxic effects of this peptide (Biéler et al., 2005). The aggregation of the peptide is modulated by many environmental factors as well as the state of posttranslational modification of the peptides.

MccE492 is produced in both the unmodified and modified forms in culture. The modified, antimicrobial MccE492 is the predominant form while the bacteria are growing in exponential phase of the culture (Marcoleta et al., 2013). When the bacteria begin to enter stationary phase, they decrease the production of the modified form making the unmodified MccE492 more prevalent in the population (Marcoleta et al., 2013). Unmodified MccE492 polymerizes faster than the modified peptide in forming amyloid fibers leading them to hypothesis that the bacteria may begin to produce more unmodified peptide in stationary phase is to begin to detoxify the environment by sequestering these peptides in inert amyloids (Marcoleta et al., 2013). Even though the unmodified form is more efficient in polymerizing, the modified MccE492 is found in fibers with the unmodified form (Marcoleta et al., 2013). Moreover, polymerization of the modified MccE492 is accelerated in the presence of unmodified seeds, small amyloids that can nucleate amyloid elongation (Marcoleta et al., 2013).

Apart from the influence of posttranslational modification on MccE492 polymerization, the environment has a profound effect of the rate of polymerization and can even cause disassembly of MccE492 amyloids (Shahnawaz and Soto, 2012). Basic pH, low ionic strength, and dilution of the fibers all led to fiber disassembly in two hours (Shahnawaz and Soto, 2012). These disassembled fibers regained their antimicrobial activity that was absent while in amyloid fibers (Shahnawaz and Soto, 2012). Even more striking was the ability of these disassembled toxins to reform amyloids when the environment was again changed (Shahnawaz and Soto, 2012). These data demonstrate that in the case of MccE492, there are many factors that influence the toxicity of the peptides suggesting that the *Klebsiella pneumonia* has evolved a mechanism of efficiently modulating the toxicity of MccE942. This data also leads to the exciting hypothesis that other amyloids may have conditions that lead to the disassembly of the amyloid fiber.

### TasA

TasA was originally described as a protein in *Bacillus subtilis* that was involved in sporulation but was later found to be expressed in stationary phase cells (Stover and Driks, 1999b,c). It has been demonstrated to have widespread antimicrobial activities against both plant and animal pathogens and commensal bacteria (Stover and Driks, 1999c). Recently though, TasA has been shown to produce amyloid fibers in *B. subtilis* biofilms that contributes heavily to the formation of complex community architecture similar to curli in *E. coli* (Romero et al., 2010). TasA is part of an operon that also encodes for TapA, the minor amyloid subunit and fiber anchor, and SipW, the signal peptidase that processes TapA and TasA (Stover and Driks, 1999a,c). It has been proposed that the antimicrobial effects of TasA may be due to the formation of toxic oligomers that many amyloidogenic proteins generate, but to our knowledge, this has not yet been investigated (Romero et al., 2010).

### LISTERIOLYSIN O

Listeriolysin O (LLO) of *Listeria monocytogenes* is a cholesterol-dependent cytolysin whose activity is dependent on pH. LLO formed pores in the membrane of phagolysosome allowing for *L. monocytogenes* to escape and carry out its replicative phase in the host cell cytosol (Portnoy et al., 1988; Cossart et al., 1989). The pH dependent activity of LLO was shown to be due to an irreversible structural change in the protein that lead to a decreased ability in hemolytic activity (Schuerch et al., 2005). Later, it was appreciated that this structural change was actually the result of LLO forming an amyloid (Bavdek et al., 2012). This pH change of the protein into an amyloid suggests that this proteins has evolved to form pores while the bacteria is trapped in a phagolysosome, but once it escapes into the cytosol, the higher pH inactivates the proteins by triggering amyloidogenesis (Bavdek et al., 2012). This amyloidogenesis may prevent the LLO toxin from lysing the infected cell while *L. monocytogenes* replicates in the host cell. It is unknown if LLO amyloids demonstrate any activity intracellularly.

### HARPINS

Harpins are a class of proteins that are produced by gram-negative plant pathogens. They are characterized by being glycine rich, heat stable proteins that are secreted by a type III secretion system that can trigger a hypersensitive response (HR) in plants and are predicted to have α-helical regions (Wei et al., 1992). Harpins trigger this HR when they are present in the intercellular space. The plant cells detect these proteins and respond using the early defense response through an apoptosis-like cell death. Pathogens lacking harpins, such as HpaG of *Xanthomonas axonopodis,* have decreased virulence (Kim et al., 2003, 2004). The mechanism by which harpins trigger HR in plants in not fully understood, but there is some data supporting harpins interacting with and disrupting cell membranes leading to depolarization (Lee et al., 2001).

In 2007, a harpin from *Xanthamonas*, HpaG, was characterized biochemically (Oh et al., 2007). This group found that under conditions that mimic plant apoplasts, HpaG formed amyloid fibers (Oh et al., 2007). A mutant of the proteins (L50P) that did not trigger HR in plants and was also unable to form amyloid fibers (Oh et al., 2007). From this, the authors suggest that the transition to amyloid fibers is an important step in triggering HR. Furthermore, harpins from other plant pathogens, *Ercinia amylovora*, and *Pseudomonas syringae* also formed amyloid fibers in plant apoplast-like conditions (Oh et al., 2007). More detailed studies on harpins are needed to determine exactly what properties can be attributed to soluble and amyloid forms of these proteins.

## AMYLOID INHIBITORS

An exciting field is emerging that is trying to speed up or slow down the formation of amyloid fibers using small molecules. The idea is that for many amyloidogenic proteins, the toxicity is due to the formation of intermediate oligomers that can disrupt membranes. By accelerating the polymerization of amyloid subunits, we may be able to bypass the toxic, degenerative affect of amyloids slowing the progression of neurodegenerative disease. Along similar lines, since many bacterial amyloids have been shown to aid in adherence to surfaces, if we use small molecules to interfere with the polymerization, bacteria may not be able to anchor themselves to the host in infections leading to faster clearance of pathogens. Conversely, in cases like the PSMs of *S. aureus*, since the soluble form is a toxin that is able to disrupt the immune response, by causing polymerization of the monomers could prevent their toxic function to cells allowing the immune system to clear the infection.

Curlicides and pilicides are ring-fused 2-pyridone molecules that are designed to look like peptide backbones. They have been designed to mimic a proteins backbone and interact with proteins that form amyloids by either disrupting their ability to polymerize, or nucleating and accelerating the amyloid maturation (Andersson et al., 2013). They have so far been characterized with their interaction with *E. coli* curli and type 1 pili (Andersson et al., 2013). These molecules are not only able to influence the *in vitro* polymerization of CsgA, but they are able to affect the biogenesis of curli and pili in *E. coli* biofilms (Andersson et al., 2013). Additionally, uropathogenic *E. coli* treated with curlicides were attenuated in a murine model of a urinary tract infection (Cegelski et al., 2009). Other groups have taken the approach of designing small, non-natural peptides that can disrupt amyloid formation (Sievers et al., 2011). This has been shown to be successful *in vitro* with disease associated amyloids (Sievers et al., 2011). Recently, TasA has been proposed to be model amyloid for screening molecules with widespread activity against amyloids necessary for biofilm formation (Romero et al., 2013). Ongoing research is increasing the efficacy of these molecules as well as characterizing their ability to modulate the biogenesis of other amyloids.

## FUNCTIONAL AMYLOIDS IN OTHER KINGDOMS OF LIFE

Eukaryotic amyloids have been the subject of many recent reviews (Liebman and Chernoff, 2012; Watt et al., 2013; Wickner et al., 2013). Importantly, functional amyloids are not exclusive to bacteria. They have been found to be important in diverse eukaryotes from yeast to humans. It has recently become appreciated that humans have several functional amyloids. The protein Pmel17, a protein made in melanosomes for mammalian pigmentation, forms amyloids fibers (Fowler et al., 2005). The production of this amyloids is highly regulated with cells utilizing proteolytic processing to mature the proteins into a form that can form amyloids (Berson et al., 2001; Kummer et al., 2009). These processing steps prevent the proteins from forming amyloids in other cellular compartments preventing the toxicity that is associated with disease-associated amyloids. Additionally, it has been found that peptide hormones that are stored in the secretory granules of the mammalian pituitary form amyloids (Maji et al., 2009). It was found that *in vitro*, 31 of 42 studied hormones were able to form amyloids fibers (Maji et al., 2009). Mouse pituitary’s also contained amyloid fibers using various approaches to identify amyloids (Maji et al., 2009). It is thought that these peptide hormones are stored in secretary granules as amyloids until they are needed. The cells can then secrete the granules and dilution of the amyloids causes disassociation and activation of the peptide hormones (Maji et al., 2009).

Many yeast form prions, self-propagating amyloids that are heritable elements. This method of non-mendelian inheritance was first proposed for [URE3] (Wickner, 1994). It was shown that the cytoplasmically inherited [URE3] element has the opposite effect on ureidosuccinate metabolism as the Ure2 protein and that cells cured of [URE3] were able to regain the element when Ure2 is overexpressed (Aigle and Lacroute, 1975; Wickner, 1994). This lead Wickner to the hypothesis that cytoplasmically inherited elements in yeast were prions (Wickner, 1994). Since then, several yeast proteins have been shown to form prions that, in most cases, abrogate the function of proteins.

## CONCLUDING REMARKS

It is becoming ever more appreciated that the amyloid fold is not just a product of protein misfolding, but it is a ubiquitously used protein fold throughout the kingdoms of life. Amyloids provide structure or control availability of proteins such as toxins or signaling molecules. Even more exciting is the discovery that some of these proteins have been found to have different functions when they are in their soluble or insoluble forms. The production of functional amyloids is a highly controlled and regulated process that is controlled on several levels including transcriptional, translational, and posttranslational. The difficulty associated with breaking up these proteins is the property that has made them so valuable for many organisms. In the case of many bacteria, these amyloids provide a structural component that keep the community protected against mechanical and enzymatic disruption (Schwartz et al., 2012). In others, they are used as reservoirs of toxins that are ready to become active once the environment changes (Shahnawaz and Soto, 2012). This field is only beginning to look at the effect that these functional amyloids play in the dynamic relationships between bacterial species as well as how these proteins may be involved in bacterial interactions with the host at a commensal or pathogenic level. It will be exciting to see where this field of bifunctional bacterial proteins goes as well as targeting these proteins to disperse biofilms or to sequester toxins.

## Conflict of Interest Statement

The authors declare that the research was conducted in the absence of any commercial or financial relationships that could be construed as a potential conflict of interest.
